# Constitutive and herbivore-induced systemic volatiles differentially attract an omnivorous biocontrol agent to contrasting *Salix* clones

**DOI:** 10.1093/aobpla/plt005

**Published:** 2013-02-05

**Authors:** Anna Lehrman, Tina Boddum, Johan A. Stenberg, Colin M. Orians, Christer Björkman

**Affiliations:** 1 Department of Crop Production Ecology, Swedish University of Agricultural Sciences, PO Box 7043, 75007 Uppsala, Sweden; 2 Department of Plant Protection Biology, Swedish University of Agricultural Sciences, PO Box 102, 23053 Alnarp, Sweden; 3 Department of Ecology, Swedish University of Agricultural Sciences, PO Box 7044, 75007 Uppsala, Sweden; 4 Department of Biology, Tufts University, Medford, MA 02155, USA

**Keywords:** Biocontrol, biological control, blue willow beetle, common flowerbug, E-4,8-dimethyl-1,3,7-nonatriene, GC electro-antennogram, Z-3-hexenyl acetate, short rotation coppice

## Abstract

While carnivores are known to be attracted to herbivore-induced plant volatiles, little is known about how such volatiles may affect the behaviour of omnivorous predators that may use both plants and herbivores as food. Here, we examine how systemically produced plant volatiles, in response to local herbivore damage, differentially attract a key omnivorous predator, *Anthocoris nemorum* (Heteroptera: Anthocoridae), to single clones of three species of *Salix*: *S. viminalis*, *S. dasyclados* and *S. cinerea*. The profiles of the plant volatiles produced were found to vary among *Salix* clones and between herbivore-damaged and intact plants. *Anthocoris nemorum* was attracted to the volatiles released from undamaged plants of all three species, but most strongly to a native *S. cinerea* clone. Plants damaged by the herbivorous leaf beetle *Phratora vulgatissima* (Coleoptera: Chrysomelidae) were generally more attractive than undamaged plants, with *A. nemorum* responding to systemic changes in the damaged plants where the experimental design specifically excluded volatiles released from the actual site of damage. When comparing damaged plants, the *S. dasyclados* clone was more attractive to *A. nemorum* than the *S. viminalis* clone—a somewhat surprising result since this *Salix* clone is considered relatively resistant to *P. vulgatissima*, and hence offers a limited amount of prey. Our experiments highlight that both constitutive and induced plant volatiles play a role in omnivore attraction, and this emphasizes the importance of considering odours of released volatiles when cropping and breeding *Salix* for increased resistance to herbivores.

## Abstract (precis)

Little is known about how herbivore-induced plant volatiles affect omnivorous predators. Here we show that the key predator *Anthocoris nemorum* is differentially attracted to three *Salix* clones when these are damaged by the detrimental blue willow beetle (*Phratora vulgatissima*). At least two volatile plant compounds were induced by the herbivore, and these were antennal active in the predator. The results elucidate how plants may recruit omnivorous predators when damaged. These findings could be utilized in crop breeding for increased resistance against herbivores.

## Introduction

The abundance of herbivores on a plant is determined directly by plant traits (bottom-up) and natural enemies (top-down), and indirectly by the effects of plant traits on the natural enemies of the herbivore (Takabayashi *et al.* 1991; Halitschke *et al*. 2000; Dicke 2009). The importance of indirect effects is currently receiving increased attention as evidence accumulates concerning the exploitation of plant volatiles, especially herbivore-induced plant volatiles (HIPVs), by natural enemies when locating their prey (Mumm and Dicke 2010). Herbivore-induced plant volatiles are released both locally, at the site of damage, and/or systemically in non-damaged leaves (Turlings and Tumlinson 1992; Halitschke *et al.* 2000). Importantly, the blend of volatiles can vary according to plant species, plant cultivar and the developmental stage of the plant, and this blend can alter the behavioural response of some natural enemies (Takabayashi and Dicke 1996). Thus, in order to make use of a herbivore's natural enemies in a biocontrol programme, it is necessary to know how the enemies respond to the volatiles released by a specific plant species or cultivar. An additional factor that may confound indirect effects is that some enemies are omnivorous. While most studies have focused on pure carnivores (e.g. Stenberg *et al.* 2007; Halitschke *et al.* 2008), recent studies have also shown that omnivorous predatory mites (Zhong *et al.* 2011) and ladybirds (Glinwood *et al.* 2009), which also gain nutrition from various plant products, respond to plant volatiles in their search for prey. Omnivores are likely to respond to both constitutive and herbivore-induced plant volatiles since, in the absence of prey, they can in some cases survive by feeding solely on plant food (Stenberg *et al.* 2010). Given the unpredictability of prey abundance, the ability of omnivores to survive on the plant even when no prey are present may make omnivores particularly suitable for biological control, provided they do not become pests themselves. Under favourable conditions, omnivorous plant ‘bodyguards’ can trigger top-down effects by suppressing herbivore populations, thereby increasing the production of plant biomass. For example, some well-studied omnivorous bugs in the genus *Geocoris* can consume lepidopteran eggs from wild tobacco, which would otherwise develop into larvae, a single one of which can consume the entire plant before any seeds are produced (Halitschke *et al.* 2008).

Willow (*Salix* spp.) is an ideal system in which to study plant–herbivore–omnivore interactions. In northern Europe the herbivorous blue willow beetle, *Phratora vulgatissima*, causes extensive damage to the native *Salix cinerea* and to exotic willows used in bioenergy production: a decrease in production of biomass by up to 40 % has been recorded (Björkman *et al.* 2000). One important predator that contributes to the regulation of this pest is the omnivorous bug *Anthocoris nemorum* (Björkman *et al.* 2003; Dalin *et al.* 2006). It is a generalist predator that is found on several plant species (Collyer 1967) and which feeds on a variety of prey including the eggs and larvae of *P. vulgatissima*. This anthocorid has also been proven capable of surviving and reproducing on *Salix* plant fluid alone (Stenberg *et al.* 2010), and previous investigators have indicated that its consumption of sap and extra-floral nectar does not compromise plant growth (e.g. Lauenstein 1979). The anthocorid can also feed on pollen and floral nectar. Thus, altogether it can utilize a wide range of plant-provided foods without damaging the plant, which makes this generalist predator interesting for biocontrol purposes. Two of the most common *Salix* species grown and bred for bioenergy in northern Europe are *S. viminalis* and *S. dasyclados* (for heritage see Kendall *et al.* 1996), of which the latter is considered to be resistant to *P. vulgatissima* and the former susceptible. The preference that *P. vulgatissima* exhibits for volatiles from different *Salix* species has been evaluated previously (Peacock *et al.* 2001).

In *Salix*, volatiles are well known to alter the composition of insect communities (Inui *et al.* 2003) and are correlated with the performance of *P. vulgatissima* (Peacock *et al.* 2001). To date, the role of plant volatiles for natural enemies of this pest has not been addressed.

In this study, we examined the attraction of *A. nemorum*, an omnivorous anthocorid predator, to plant volatiles released from three contrasting *Salix* clones that differ in suitability to *P. vulgatissima*. We used single representative clones of three species of *Salix: S. viminalis*, *S. dasyclados* and *S. cinerea*. We expected the preference to mimic the preference of *P. vulgatissima*, since this would hypothetically increase the omnivore's likelihood of finding animal food. We also examined whether induced volatiles (HIPVs) altered anthocorid preferences. Overall, we addressed the following specific questions: (i) Is *A. nemorum* attracted to constitutively produced *Salix* volatiles? (ii) Can *A. nemorum* differentiate between these *Salix* clones? (iii) Do HIPVs in *Salix* affect *A. nemorum*'s preference for particular *Salix* clones? (iv) Do the volatile profiles of damaged and undamaged *Salix* plants differ?

## Methods

### Plants

We used three *Salix* clones: the commercial clones ‘Jorunn’ (*S. viminalis*) and SW901290 (*S. dasyclados*), and one haphazardly selected clone of the native *S. cinerea*. The two commercial clones were chosen because they, like other clones of these species (I. Åhman, unpubl. data), are known to differ in their susceptibility to *P. vulgatissima*. About 6 weeks before the tests were conducted, winter cuttings (10 cm) of each clone were planted in 11-cm pots containing Hasselfors (Örebro, Sweden) planting soil. The plants were grown in greenhouses under natural light, at 18–24 °C, and continuously provided with water containing fertilizer in solution (N–P–K : 51–10–43).

### Behavioural assays

**Fig. 1:**
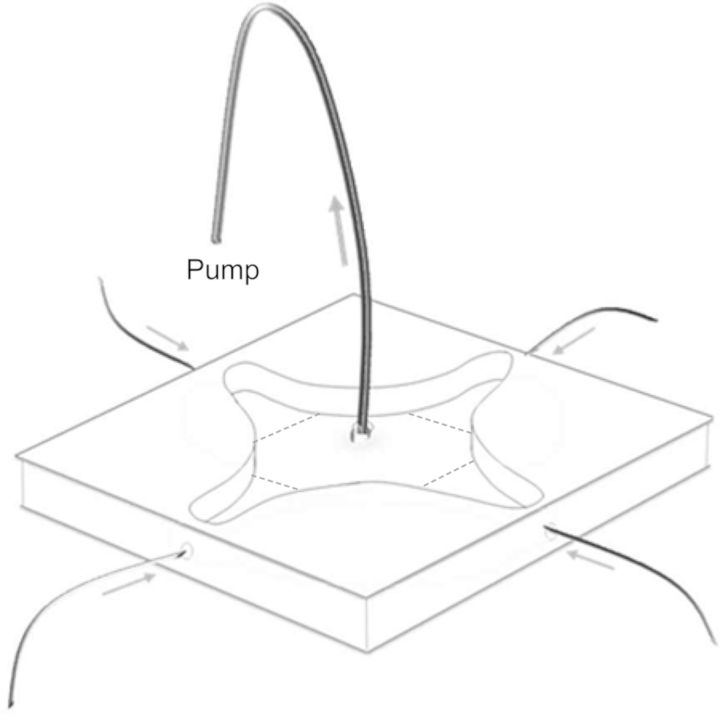
**Four-arm olfactometer (without odour sources shown) after an original design that was first presented in** Pettersson (1970). Air is sucked from the centre, thereby drawing air up the arms from odour sources, which may include isolated plants, a pot with soil or untreated air.

Behavioural assays were conducted in late May or early June in a four-arm olfactometer (Fig. 1) (Pettersson 1970). This guarantees observed ‘preferences’ to be positive choices rather than the avoidance of an odour, which can be the case when two-way arenas or Y-tubes are used. Adult female *A. nemorum* were collected from nettles (*Urtica dioica*) in the field and stored at 8 °C for 1–4 days in plastic tubes containing green beans as a neutral source of food and moisture. Before being tested, individual bugs were isolated in Eppendorf tubes at room temperature and starved for 1 h. Each plant was isolated in a 5-l baking bag (Toppits^®^, Melitta, Minden, Germany) sealed to the top of the pot with a rubber band, leaving a small gap to allow air to flow into the base and out from the upper corner of the bag, from which point a Teflon tube led to the olfactometer. A weak airflow (100 mL min^−1^), from each treatment, entered each of the four arms of the olfactometer and flowed towards the centre of the olfactometer where the air was exhausted. To ensure that there was no volatile carryover between treatments, the Teflon tubes that connected the plant bags to the olfactometer were heated to 150 °C for 3h before re-use.

The out-flow pipe was temporarily removed from the apparatus to allow individual specimens of *A. nemorum* to be released at the centre of the olfactometer. The olfactometer was divided into four choice zones and a neutral central zone in which the presence of an animal was defined as it exhibiting no choice. The location of each *A. nemorum* was recorded every third minute over a period of 30 min. The ‘visitation rate’ for each of the four choice zones was defined as ‘the number of recorded insect observations in the choice zone divided by the total number of observations’. Between each test the olfactometer was washed in detergent and 70 % ethanol, and the position of the treatments randomly re-allocated. After seven insects had been tested the plants were exchanged for new ones.

We performed three sets of experiments. In the first set, we tested whether the anthocorid was attracted to *Salix* at all, and if so, whether it could discriminate between the three contrasting clones. This was accomplished by comparing the anthocorids' attraction to airflow from undamaged *S. viminalis*, *S. dasyclados* and *S. cinerea* clones placed at the three olfactometer arms, with ambient air supplied at the fourth arm as a control.

The second set of experiments comprised two parts focusing on the *S. viminalis* and *S. dasyclados* clones. The reason why we excluded one plant was that we wanted to include both air and soil volatiles as independent controls. Therefore, the four-armed olfactometer only allowed for two more odour sources, and the *S. viminalis* and *S. dasyclados* clones were chosen because they are used in commercial plantations, and included in several ongoing research projects throughout Europe. First, we tested *A. nemorum*'s attraction to volatiles from undamaged plants and then to plants damaged by herbivory. The two plant treatments occupied two of the olfactometer's arms, with the third arm supplying airflow from a pot containing only moist soil, and the fourth supplying only ambient air. When testing plants damaged by herbivores, three starved adult *P. vulgatissima* were isolated in a 10 cm × 5 cm frying bag (Toppits^®^, Melitta, Germany) at the top of one of the shoots of the plants within the larger bag. All plants were checked to ensure that the beetles had started to feed on the leaves 1h prior to the test. The beetles were kept isolated on the shoots throughout the test. The bag enclosing the beetles prevented any volatiles from being emitted, thus ensuring that the anthocorid could only respond to systemically induced changes in the host plant. Finally, we directly compared damaged and undamaged plants within each species to determine the relative attraction of anthocorids to volatiles released from damaged and undamaged individuals (damaged plants were fed on by *P. vulgatissima* as described above). This test used only ambient air as the control because the previous results had already demonstrated that there was no significant difference between soil and air controls.

### Volatile profiling

#### Collections of headspace volatiles

Odour collection was performed over a period of 24 h using the same general set-up as for the olfactometer test, except that bags did not include pots, i.e. there were no soil volatiles, and inflowing air was pushed through activated charcoal at 600 mL min^−1^ at the base of the plants. The volatiles were collected in (Porapak Q filters, Sigma-Aldrich, St Louis, MO) placed at the top of the bags connected to a pump pulling at 300 mL min^−1^. The positive-pressure venting prevented any contamination by volatiles from external sources. Three collections from the different clones and treatments were made in parallel. Every sample was replicated six times. Six control samples, using just the bag, were also collected. Porapak Q filters were baked at 180 °C and charcoal filters and tubes were baked at 150 °C overnight under a constant stream of nitrogen before every collection. The frying bags were also baked overnight. Volatiles were extracted from filters by washing with 500 µL of hexane (high-performance liquid chromatography grade) and stored at −20 °C until analysed.

#### Chemical analysis of headspace samples

The collected odours were analysed with a gas chromatograph coupled to a mass spectrometer (GC-MS; 6890GC and 5975MS, Agilent, Santa Clara, CA). The GC-MS was equipped with a DB-wax (PEG) column and used helium as a carrier gas. A 2-µL sub-sample from each collected odour sample was injected either manually or by the autoinjector (7683B Series injector, Agilent) (for more details see Karlsson *et al*. 2009). The inlet temperature was 200 °C and the oven temperature was set to 30 °C for 3 min, and then increased by 8 °C per minute until 225 °C was reached and held for 3 min. The protocol was terminated by a 1-min post-run at 250 °C. Compounds were tentatively identified by comparing the mass spectra and retention times of the peaks in the odour collections with those from synthetic and authentic standards, and comparing with published Kovats retention indices from the same type of column or a column with similar properties.

### Data analysis

All behavioural data were analysed using SAS 9.1. Friedman analysis of variance (ANOVA) was used to test for differences in preference in each experiment. Because the variables were related (i.e. the number of observed visitations to a specific choice zone depended on the number of visitations to other choice zones), a new variable was created from the difference between the number of visits (1–10) for each individual insect and pair-wise comparison (for example *S. viminalis* vs. *S. dasyclados*) which was then tested for deviation from zero (cf. Heisswolf *et al*. 2007). These data were not normally distributed and were therefore analysed with the non-parametric Wilcoxon signed tests. The released amounts of compounds 1–5 from undamaged and damaged *S. dasyclados* were compared using a two-sample *t*-test.

## Results

**Fig. 2:**
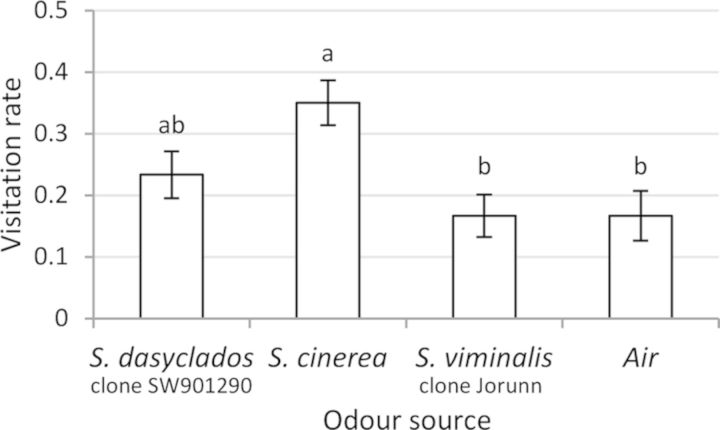
**Visitation rates (mean ± SE) of *A. nemorum* to each of four choice zones in an olfactometer during a 30-min observation period**. The four odour sources infiltrating the choice zones were volatiles from undamaged *S. viminalis*, *S. dasyclados* and *S. cinerea*, and ambient air. Different letters indicate significant differences (Wilcoxon's signed test, *P* < 0.05).

The anthocorids showed a higher preference (visitation rate) for volatiles from the undamaged *S. cinerea* clone compared with volatiles from the undamaged *S. viminalis* clone (Table 1; Fig. 2; *P* = 0.0074) or ambient air. Anthocorids' preference for volatiles of the undamaged *S. dasyclados* clone did not differ statistically from that for either *S. cinerea* or *S. viminalis* clones (*P* = 0.066 and 0.31, respectively).

**Table 1:** **Results from the Friedman ANOVA tests on *A. nemorum:* plant preference, herbivore-induced effects between *Salix* clones and herbivore-induced effects within *Salix* clones**. Statistically significant *P* values are in bold.

Friedman ANOVA		*n*	χ^2^	df	*P*
Plant preference *S. dasyclados*, *S. viminalis*, *S. cinerea* and air		18	10.64	3	**0.014**
Herbivore-induced effect—between species *S. dasyclados*, *S. viminalis*, soil and air	No herbivory	40	21.14	3	**<0.0001**
	Herbivory	15	13.21	3	**0.0042**
Herbivore-induced effect—within species No herbivory, herbivory and air × 2	*S. dasyclados*	19	4.99	2	0.083
	*S. viminalis*	17	7.63	2	**0.022**

**Fig. 3:**
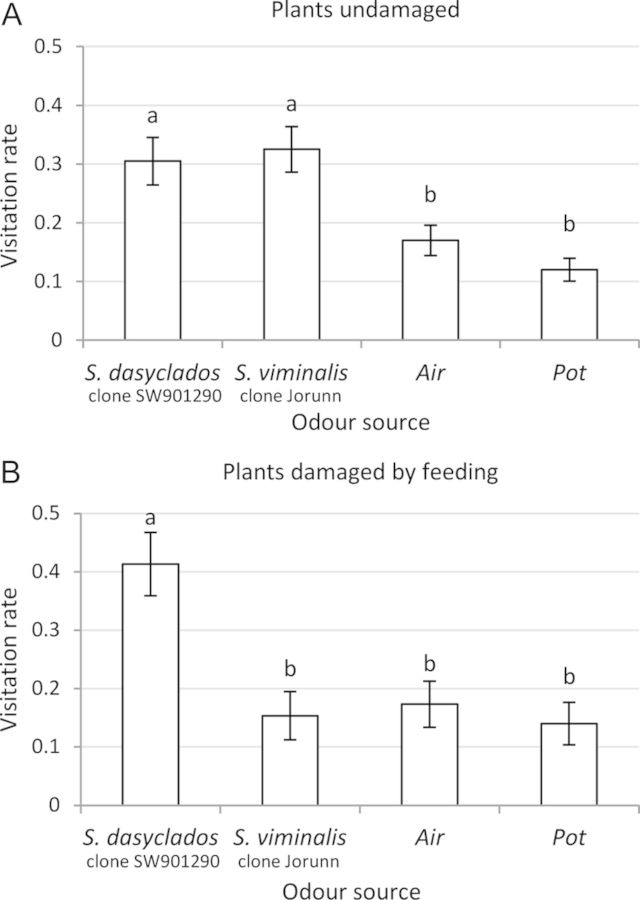
**Visitation rates (mean ± SE) of *A. nemorum* to each of four choice zones in an olfactometer during a 30-min observation period**. The four odour sources infiltrating the choice zones were (A) *n* = 40 undamaged *S. viminalis* and *S. dasyclados* with ambient air and soil as negative controls; (B) *n* = 15 *S. viminalis* and *S. dasyclados* plants subjected to herbivory by *P. vulgatissima*, with ambient air and soil as negative controls. Different letters indicate significant differences (Wilcoxon's signed test, *P* < 0.05).

**Fig. 4:**
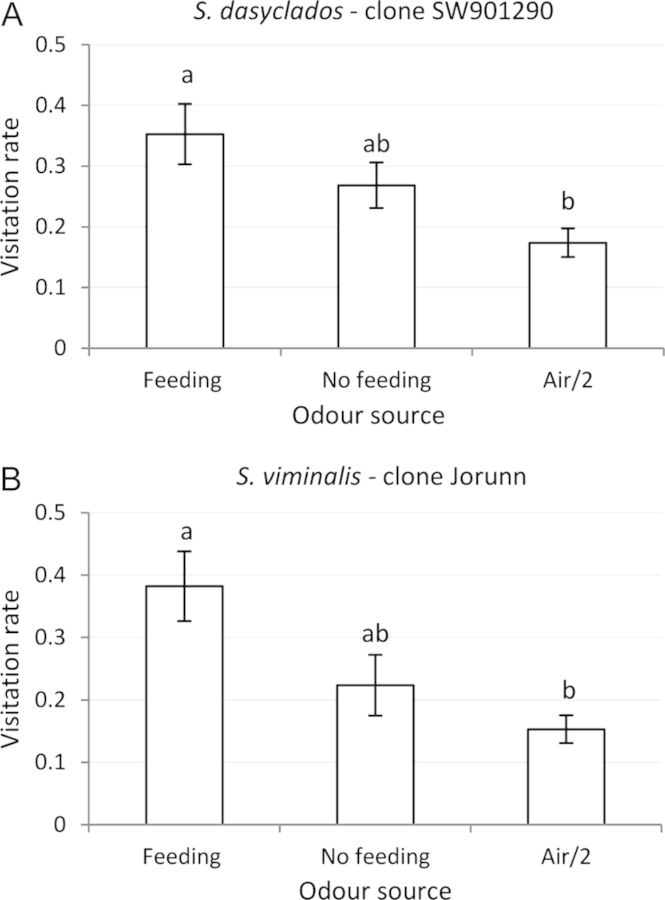
**Visitation rates (mean ± SE) of *A. nemorum* to each of four choice zones in an olfactometer during a 30-min observation period**. The four odour sources infiltrating the choice zones were: (A) herbivore (*P. vulgatissima*)-damaged *S. viminalis* and undamaged *S. viminalis*, and two ambient air zones; (B) herbivore-damaged *S. dasyclados* and undamaged *S. dasyclados*, and two ambient air zones. Different letters indicate significant difference (Wilcoxon's signed test, *P* < 0.05).

In the choice between undamaged *S. dasyclados* and *S. viminalis*, the anthocorids did not prefer one clone over the other (Table 1; Fig. 3A), but they did show a clear preference for the plants over bare soil or air (*S. viminalis P* < 0.0001 and 0.0094; *S. dasyclados P* = 0.002 and 0.0039, respectively). When the plants were subjected to herbivory, the anthocorids showed a clear preference for the *S. dasyclados* clone over the *S. viminalis* clone (Table 1; Fig. 3B; *P* = 0.0083). When the effect of herbivory, was compared within each species, there was no significant discrimination between damaged and undamaged plants. However, for the *S. viminalis* and *S. dasyclados* clones, there were indications that the anthocorid was attracted more to herbivore-damaged plants (Table 1; Fig. 4A and B).

**Fig. 5:**
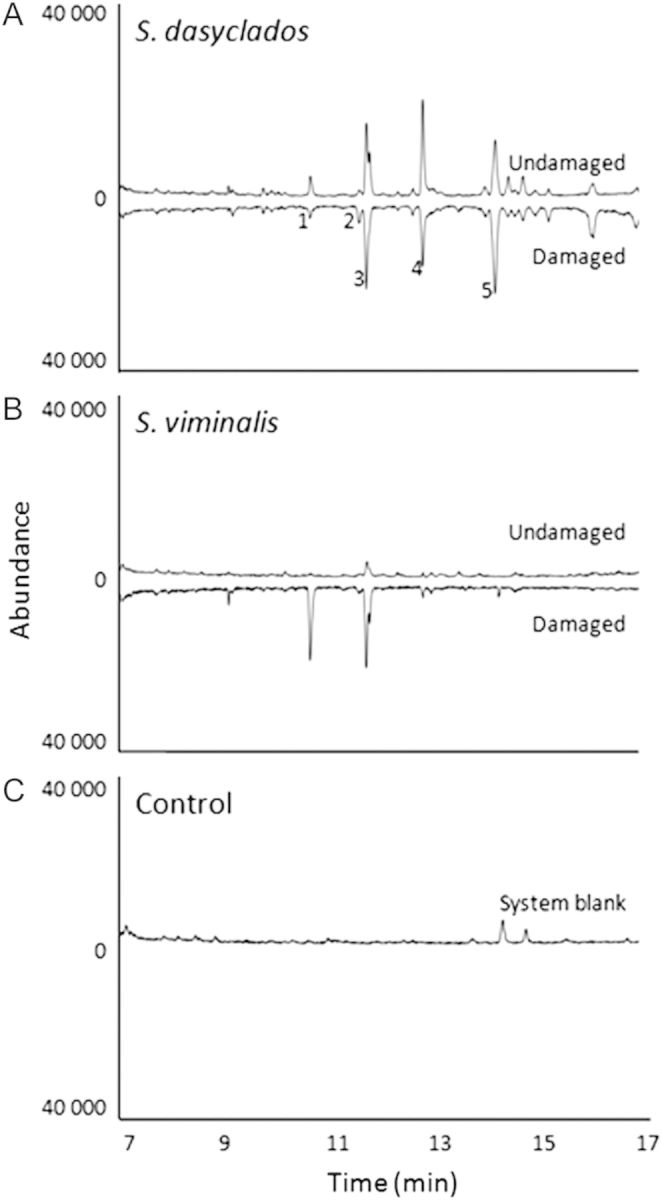
**(A) Odour profiles of undamaged and herbivore (*P. vulgatissima*)-damaged *S. dasyclados***. (B) Odour profiles of undamaged and damaged *S. viminalis*. (C) The system for odour collection without plants (control collections). The following compounds were tentatively identified from the headspace of damaged SW901290: (1) 3-carene; (2) E-4,8-dimethyl-1,3,7-nonatriene; (3) Z-3-hexenyl acetate; (4) Z-3-hexen-ol; (5) Z-3-hexenyl isovalerate.

The odour profiles of the *S. viminalis* and *S. dasyclados* clones differed (Fig. 5) and herbivory clearly altered the profiles. The control sample was clean except for the presence of silicone at the beginning of the trace (from the filters, etc.). Five compounds were tentatively identified from *S. dasyclados* headspace: 3-carene; E-4,8-dimethyl-1,3,7-nonatriene; Z-3-hexenyl acetate; Z-3-hexen-ol; and Z-3-hexenyl isovalerate. The amount of the five compounds varied but the ratio was stable [see Additional Information]. The release of E-4,8-dimethyl-1,3,7-nonatriene and Z-3-hexenyl acetate was significantly higher in damaged plants compared with undamaged plants (*P* = 0.04 and 0.05, respectively). The control sample was clean except for the presence of silicone at the beginning of the trace (from the filters, etc.).

## Discussion

Overall, we have shown that *A. nemorum*, an important omnivorous biocontrol agent in *Salix* plantations, is differentially attracted to several clones of undamaged *Salix*, and that, furthermore, *A. nemorum*'s preferences for different clones changed when there was a genotype-specific systemic change in the volatile profile induced by *P. vulgatissima* feeding on the plants. In contrast to our predictions, the host plant preference of *A. nemorum* did not mirror the preference of its main prey, the pest insect *P. vulgatissima*. While the latter is less attracted to, and performs worse on, *S. dasyclados* relative to other *Salix* species (Kendall *et al.* 1996; Glynn *et al.* 2004; Peacock *et al.* 2004), *A. nemorum* was attracted to volatiles emitted by the *S. dasyclados* clone, both when damaged and when undamaged.

More specifically, the three *Salix* clones differed in their attraction to *A. nemorum*; the *S. cinerea* clone was significantly more attractive than the *S. viminalis* clone while the *S. dasyclados* clone did not differ from the other two. Interestingly, when the plants suffered feeding damage by *P. vulgatissima*, the *S. dasyclados* clone was preferred. This pattern may result in attraction to a host that supports a few herbivores and thus could be a maladapted response to *S. dasyclados*, which is not native to Sweden. On the other hand, Stenberg *et al.* (2010, 2011) have recently shown that *S. dasyclados* provides high-quality plant food to *A. nemorum*, and propose a plant-centred hypothesis suggesting that the high-quality plant food provided by *S. dasyclados* may be important in supporting *A. nemorum* as part of an indirect defence against detrimental *P. vulgatissima* herbivores. Using the same plant-centred perspective we find it intriguing that the *S. dasyclados* clone used here is also highly attractive to *A. nemorum* when herbivore damaged.

Herbivore-induced volatiles are known to differ in both quality and quantity among plant genotypes (Dicke and Baldwin 2010). We found that *P. vulgatissima* feeding induced changes in the odour profile of both *S. dasyclados* and *S. viminalis*. The compounds released from damaged *S. dasyclados* headspace are common plant compounds (Ruther 2000; Matsui 2006) known to be involved in plant–insect interactions (Heil 2010).

A pilot GC electro-antennogram study on *A. nemorum* antennal responses has demonstrated that three of the compounds released from damaged *S. dasyclados* (3-carene, E-4,8-dimethyl-1,3,7-nonatriene and Z-3-hexenyl acetate) were antennally active (J. A. Stenberg *et al*., unpubl. data). Predatory bugs have previously been shown to utilize terpenoids and ‘green leaf volatiles’ to locate their prey on herbivore-attacked plants (Halitschke *et al.* 2008). These compounds are known to be emitted in response to herbivore attack (Takabayashi *et al.* 1991) and are attractive to both generalist and specialist predators.

## Conclusions and forward look

There is an impending risk for pest insects to overcome plants' resistance to them if, or when, plants with strong resistance become more common in plantations (cf. Panda and Khush 1995). This makes natural enemies especially important, not only for their direct ability to regulate pest numbers, but also for their role in preventing the evolution of new resistant pest populations. Previous studies have shown that *A. nemorum* is relatively insensitive to phenolic glycosides that constitute a cornerstone in the direct defence in *Salix* (Rank *et al*. 1998; Stenberg *et al.* 2010, 2011). Thus, potential conflicts between direct and indirect defences seem to be minimal in *Salix*. The fact that the omnivorous predator *A. nemorum* is a widespread and common species makes it an especially important player in the *Salix* system, even though its importance varies between sites (Gross *et al.* 2004). The fact that resistant and moderately resistant *Salix* clones are more efficient in attracting *A. nemorum* than the susceptible clone strengthens our plant-centred defence theory and opens up novel opportunities for improved breeding. The fact that *S. dasyclados* is both more resistant to the herbivorous beetles and more attractive to the predator suggests that species such as these are likely to be highly suitable for bioenergy production. In summary, the results presented here lead us one step closer towards understanding the bottom-up and top-down regulators of *P. vulgatissima* populations in *Salix* plantations. How these two regulators interact to shape the overall pest resistance of plants constitutes an important question for future research.

## Additional information

The following additional information is available in the online version of this article –

File 1: Table showing the relative amounts (ion intensities) of the compounds released from undamaged and damaged *S. dasyclados*.

## Sources of funding

This work was funded by The Swedish Energy Agency, The Faculty of Natural Resources and Agricultural Sciences at SLU, and Lantmännen Agroenergi AB through the SAMBA project.

## Contributions by the authors

All authors planned the study and wrote the manuscript. A.L. carried out the behavioural assays and analysed the resulting data. T.B. carried out the volatile profiling and analysed the resulting data.

## Conflicts of interest statement

None declared.

## Supplementary Material

Additional Information
