# Supplementary material for: Constitutive and herbivore-induced systemic volatiles differentially attract an omnivorous biocontrol agent to contrasting Salix clones
Source: AoB Plants. 2013 Feb 5;5:plt005. doi: 10.1093/aobpla/plt005 (PMC3587182; doi:10.1093/aobpla/plt005)
Supplement: Additional Information [file supp_5_plt005_index.html]

Constitutive and herbivore-induced systemic volatiles differentially attract an omnivorous biocontrol agent to contrasting Salix species — Constitutive and herbivore-induced systemic volatiles differentially attract an omnivorous biocontrol agent to contrasting Salix clones — Additional Information 

# Constitutive and herbivore-induced systemic volatiles differentially attract an omnivorous biocontrol agent to contrasting *Salix* clones

## Additional Information

**Files in this Data Supplement:**

- Additional Information - docx file
