# Supplementary material for: Constitutive and herbivore-induced systemic volatiles differentially attract an omnivorous biocontrol agent to contrasting Salix clones
Source: AoB Plants. 2013 Feb 5;5:plt005. doi: 10.1093/aobpla/plt005 (PMC3587182; doi:10.1093/aobpla/plt005)
Supplement: Additional Information [file supp_plt005_plt005supp.docx]

|  |  |  | |
| --- | --- | --- | --- |
| **Realease from *Salix dasyclados*** | | | |
| (Avg peak area/1000 (±SEM)) | | | |
|  |  | *Undamaged* | *Damaged* |
| Compound | 1 | 126 (±75) | 215 (±148) |
|  | 2 | 60 (±22)* | 156 (±26) |
|  | 3 | 577 (±134)* | 961 (±71) |
|  | 4 | 1418 (±71) | 837 (±315) |
|  | 5 | 1162 (±262) | 1672 (±565) |
| **Ratio** | | **2:1:10:23:19** | **1:1:6:5:10** |
|  |  |  |  |

**Table S1 Relative amounts (ion intensities) of the compounds released from undamaged and damaged *Salix dasyclados.*** The average amounts (±SEM) and the ratio between the compounds are stated. All odor collections were repeated 3 times. For compounds marked with * there were significant differences in the released amounts from undamaged and damaged plants.
